# Automated DWI-FLAIR mismatch assessment in stroke using DWI only

**DOI:** 10.1093/esj/23969873251362712

**Published:** 2026-01-01

**Authors:** Joseph Benzakoun, Lauranne Scheldeman, Anke Wouters, Bastian Cheng, Martin Ebinger, Matthias Endres, Jochen B Fiebach, Jens Fiehler, Ivana Galinovic, Keith W Muir, Norbert Nighoghossian, Salvador Pedraza, Josep Puig, Claus Z Simonsen, Vincent Thijs, Götz Thomalla, Emilien Micard, Bailiang Chen, Bertrand Lapergue, Grégoire Boulouis, Alice Le Berre, Jean-Claude Baron, Guillaume Turc, Wagih Ben Hassen, Olivier Naggara, Catherine Oppenheim, Robin Lemmens

**Affiliations:** IMA-BRAIN, INSERM U1266, Institute of Psychiatry and Neuroscience of Paris (IPNP), Université Paris Cité, Paris, France; Neuroradiology Department, Hôpital Sainte Anne, GHU-Paris Psychiatrie et Neurosciences, Paris, France; Department of Neurosciences, Experimental Neurology, KU Leuven - University of Leuven, Leuven, Belgium; Department of Neurosciences, Experimental Neurology, KU Leuven - University of Leuven, Leuven, Belgium; Department of Neurology, University Hospitals Leuven, Leuven, Belgium; Department of Neurosciences, Experimental Neurology, KU Leuven - University of Leuven, Leuven, Belgium; Department of Neurology, University Hospitals Leuven, Leuven, Belgium; Department of Neurology, University Medical Center Hamburg-Eppendorf, Hamburg, Germany; Department of Neurology, Medical Park Berlin Humboldtmühle, Berlin, Germany; Department of Neurology, Charité – Universitätsmedizin Berlin, Berlin, Germany; Center for Stroke Research Berlin, Charité – Universitätsmedizin Berlin, Berlin, Germany; Department of Diagnostic and Interventional Neuroradiology, University Medical Center Hamburg-Eppendorf, Hamburg, Germany; Center for Stroke Research Berlin, Charité – Universitätsmedizin Berlin, Berlin, Germany; School of Cardiovascular & Metabolic Health, University of Glasgow, Glasgow, United Kingdom; Hospices Civils de Lyon, Department of Neurology and Stroke Center, Lyon University, Lyon, France; Université Claude Bernard Lyon 1, Bron, France; Department of Radiology (CDI) and IDIBAPS, Hospital Clinic, Barcelona, Spain; Department of Radiology (CDI) and IDIBAPS, Hospital Clinic, Barcelona, Spain; Department of Neurology, Aarhus University Hospital, Aarhus, Denmark; Stroke Division, University of Melbourne, Florey Institute of Neuroscience and Mental Health, Melbourne, VIC, Australia; Department of Neurology, University Medical Center Hamburg-Eppendorf, Hamburg, Germany; CIC, Innovation Technologique, Université de Lorraine, INSERM 1433, Nancy, France; CIC, Innovation Technologique, Université de Lorraine, INSERM 1433, Nancy, France; Department of Neurology, Foch Hospital, Versailles Saint-Quentin-en-Yvelines University, Suresnes, France; Diagnostic and Interventional Neuroradiology Department, CHRU de Tours, Tours, Centre Val de Loire, France; CIC-IT 1415, INSERM 1253 iBrain, Tours, Centre Val de Loire, France; IMA-BRAIN, INSERM U1266, Institute of Psychiatry and Neuroscience of Paris (IPNP), Université Paris Cité, Paris, France; Neuroradiology Department, Hôpital Sainte Anne, GHU-Paris Psychiatrie et Neurosciences, Paris, France; Stroke Team, INSERM U1266, Institute of Psychiatry and Neuroscience of Paris (IPNP), Université Paris Cité, Paris, France; Neurology Department, Hôpital Sainte Anne, GHU-Paris Psychiatrie et Neurosciences, Paris, France; Stroke Team, INSERM U1266, Institute of Psychiatry and Neuroscience of Paris (IPNP), Université Paris Cité, Paris, France; Neurology Department, Hôpital Sainte Anne, GHU-Paris Psychiatrie et Neurosciences, Paris, France; IMA-BRAIN, INSERM U1266, Institute of Psychiatry and Neuroscience of Paris (IPNP), Université Paris Cité, Paris, France; Neuroradiology Department, Hôpital Sainte Anne, GHU-Paris Psychiatrie et Neurosciences, Paris, France; IMA-BRAIN, INSERM U1266, Institute of Psychiatry and Neuroscience of Paris (IPNP), Université Paris Cité, Paris, France; Department of Neurosciences, Experimental Neurology, KU Leuven - University of Leuven, Leuven, Belgium; IMA-BRAIN, INSERM U1266, Institute of Psychiatry and Neuroscience of Paris (IPNP), Université Paris Cité, Paris, France; Neuroradiology Department, Hôpital Sainte Anne, GHU-Paris Psychiatrie et Neurosciences, Paris, France; Department of Neurosciences, Experimental Neurology, KU Leuven - University of Leuven, Leuven, Belgium; Department of Neurology, University Hospitals Leuven, Leuven, Belgium

**Keywords:** Ischemic stroke, magnetic resonance imaging, diffusion magnetic resonance imaging, artificial intelligence, decision support techniques

## Abstract

**Introduction:**

In Acute Ischemic Stroke (AIS), mismatch between Diffusion-Weighted Imaging (DWI) and Fluid-Attenuated Inversion-Recovery (FLAIR) helps identify patients who can benefit from thrombolysis when stroke onset time is unknown (15% of AIS). However, visual assessment has suboptimal observer agreement. Our study aims to develop and validate a Deep-Learning model for predicting DWI-FLAIR mismatch using solely DWI data.

**Patients and methods:**

This retrospective study included AIS patients from ETIS registry (derivation cohort, 2018–2024) and WAKE-UP trial (validation cohort, 2012–2017). DWI-FLAIR mismatch was rated visually. We trained a model to predict manually-labeled FLAIR visible areas (FVA) matching the DWI lesion on baseline and early follow-up MRIs, using only DWI as input. FVA-index was defined as the volume of predicted regions. Area under the ROC curve (AUC) and optimal FVA-index cutoff to predict DWI-FLAIR mismatch in the derivation cohort were computed. Validation was performed using baseline MRIs of the validation cohort.

**Results:**

The derivation cohort included 3605 MRIs in 2922 patients and the validation cohort 844 MRIs in 844 patients. FVA-index demonstrated strong predictive value for DWI-FLAIR mismatch in baseline MRIs from the derivation (*n* = 2453, AUC = 0.85, 95%CI: 0.84–0.87) and validation cohort (*n* = 844, AUC = 0.86, 95%CI: 0.84–0.89). With an optimal FVA-index cutoff at 0.5, we obtained a kappa of 0.54 (95%CI: 0.48–0.59), 70% sensitivity (378/537, 95%CI: 66–74%) and 88% specificity (269/307, 95%CI: 83–91%) in the validation cohort.

**Discussion and conclusion:**

The model accurately predicts DWI-FLAIR mismatch in AIS patients with unknown stroke onset. It could aid readers when visual rating is challenging, or FLAIR unavailable.

## Graphical Abstract

**Graphical abstract:**
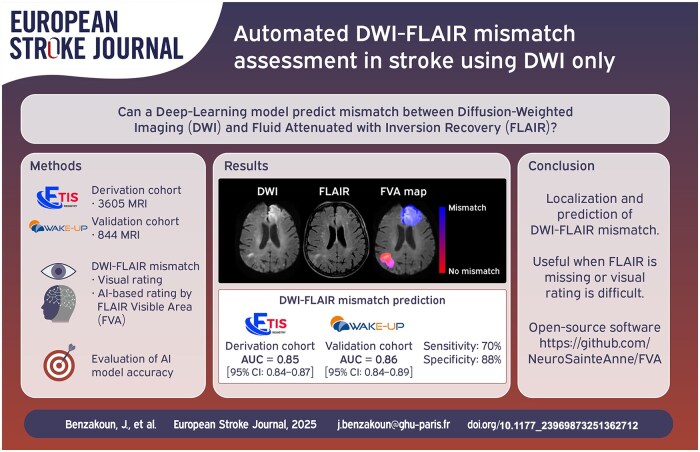

## Introduction

Acute Ischemic Stroke (AIS) is a major cause of mortality and disability worldwide, and its incidence is projected to increase in the near future.^1^ Prompt diagnosis is crucial, as each minute gained before initiation of treatment enhances patient outcome.^2^ Treatment with intravenous thrombolysis (IVT), which aims at dissolving the clot responsible for arterial occlusion, has traditionally been limited to patients with stroke onset within 4.5h, in order to minimize the risk of secondary hemorrhage.^3^ However, stroke onset time is unknown in 15% of stroke patients ^4^; in these situations, MRI findings can be used as a “tissue clock” to guide treatment decision.^5^ In the acute phase of stroke, ischemic lesions appear rapidly as hyperintensities on Diffusion-Weighted Imaging (DWI), while Fluid-Attenuated Inversion Recovery (FLAIR) changes typically occur after a few hours, in the early subacute phase of stroke.^6^ The “DWI-FLAIR mismatch” – characterized by a positive DWI lesion without obvious FLAIR hyperintensity in the corresponding area – serves as a biomarker for estimating presentation within the 4.5 h time window^5^ and identify patients with unknown stroke onset time who benefit from IVT according to the multicenter, randomized WAKE-UP trial.^7^ However, the main limitation of the DWI-FLAIR mismatch visual assessment is its low intra- and inter-observer reliability.^5,8^ Attempts at quantifying DWI and/or FLAIR with relative intensities have been proposed as an alternative, but without clear added value as compared to visual estimation.^9,10^

Recently, Artificial Intelligence (AI) algorithms aimed to address this challenge, by predicting onset-to-imaging delay based on DWI and FLAIR^11–17^ rather than detection of DWI-FLAIR mismatch. While these approaches rely on the established correlation between DWI-FLAIR mismatch and onset-to-imaging delay,^5,18^ treatment decision for unknown onset stroke patients has been validated on DWI-FLAIR mismatch, not on estimated delay, raising questions about the translation of these algorithms to clinical practice. An algorithm tailored for DWI-FLAIR mismatch detection may thus have better acceptability than those predicting onset-to-imaging delay, as it aligns more closely with the established clinical criteria used in decision-making processes.

Previous studies have proposed to compute a generative FLAIR sequence based on the DWI sequence, suggesting that DWI alone contains information relevant to DWI-FLAIR mismatch.^19–21^ Using only DWI for DWI-FLAIR mismatch prediction offers several advantages: it allows for anticipating the treatment decision as soon as the DWI is available in scenarios where timely decision-making is crucial, and should facilitate the evaluation of DWI-FLAIR mismatch in restless patients, in whom FLAIR might be non-assessable or missing.

Our study develops and validates a Deep-Learning model that performs a regional prediction of DWI-FLAIR mismatch, using solely DWI data. This model is trained on MRI obtained in AIS patients within a large derivation cohort and performance validated in the derivation cohort and in an independent dataset issued from the WAKE-UP trial.

## Materials and methods

### Data sources

**Figure 1.**
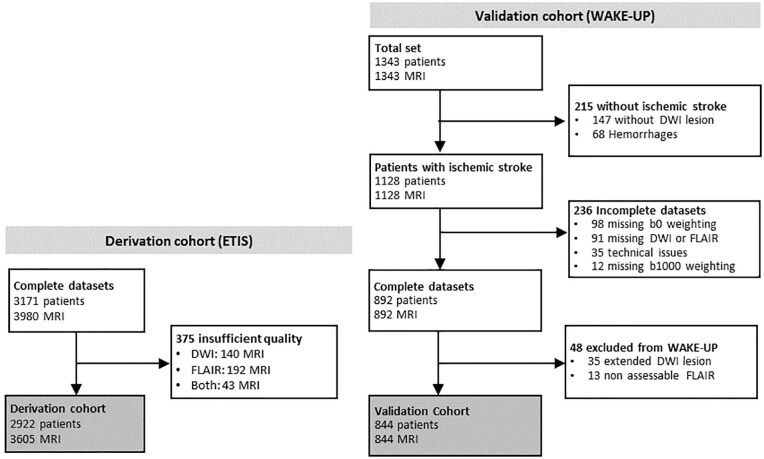
Flowchart for derivation and validation cohorts. “Extended DWI lesions” corresponded to lesions larger than 2/3 of middle cerebral artery territory (according to WAKE-UP trial inclusion criteria); “Non assessable FLAIR” included low-quality FLAIR. In the derivation cohort, in contrast to the validation cohort, the availability of sequences was checked before inclusion and all subjects had an acute ischemic stroke.

This retrospective study included two sources (Figure 1). The derivation cohort, from the Endovascular Treatment in Ischemic Stroke (ETIS) registry (ClinicalTrial.gov: NCT03776877), included adult AIS patients screened for reperfusion therapy between 2017 and 2024. When available, early follow-up MRIs were included to increase the rate of MRs without DWI-FLAIR mismatch, underrepresented in the ETIS cohort. Eligible patients had available baseline (day-0) ± early follow-up (day-1) MRI, with FLAIR and DWI sequences with *b* = 0 and *b* = 1000 s/mm^2^ weightings. Insufficient FLAIR or DWI quality was an exclusion criterion. The validation cohort was derived from the prospective multicentric, international WAKE-UP Stroke Trial (ClinicalTrials.gov NCT01525290) conducted between 2012 and 2017. We included all adult patients who underwent MRI screening for AIS with unknown onset-time, and excluded: Missing DWI and/or FLAIR; missing b0 or b1000 DWI-weightings; technical issues with raw data; subjects with exclusion criteria (hemorrhagic strokes, FLAIR rating impossible, infarct larger that >2/3 middle cerebral artery, no DWI lesion).^7,22^ Since randomization was performed after imaging in the WAKE-UP Stroke Trial, we included all screened patients, including randomized (selected based on DWI-FLAIR mismatch) and non-randomized patients.

For both cohorts, clinical data (age, sex, National Institutes of Health Stroke Scale at admission, treatment, onset delay when known) were collected. DWI lesion volumes were automatically measured using ADSv1.3.^23^

The study was approved by the Ethics Committee of UZ Leuven, Belgium under the number S68852. Patients gave written informed consent as participants in the ETIS registry and the WAKE-UP Stroke trial.

### Derivation cohort rating

Before model training, one reader (JB, neuroradiologist with 7-year experience) evaluated image quality as sufficient or insufficient. The reader then roughly contoured DWI lesion area (“infarct region”) using a large selection tool. After delineation, the reader then determined within each region if FLAIR hyperintensity was clearly visible as per WAKE-UP trial specifications,^22^ thus defining the “FLAIR Visible Areas” (FVA). MRIs were labeled as “DWI-FLAIR mismatch” if no FVA was present.

### Validation cohort rating

For patients randomized in the WAKE-UP Stroke Trial, DWI-FLAIR mismatch labeling was based on final image review board (IRB) labeling.^22^ For non-randomized patients, DWI-FLAIR mismatch was defined by using existing IRB labelings available in WAKE-UP Stroke Trial data, or by an additional labeling in consensus (JB and LS, neurologist with 3-year experience), whenever IRB labelings where not available.

In the WAKE-UP trial, patients were randomized based on DWI-FLAIR mismatch assessment in each center defined as “on-site assessment,” which did not always match the final rating. The performance of this “on-site assessment,” reflecting clinical practice, was compared to that of Deep learning model using final DWI-FLAIR labeling as gold standard.

Lacunar infarcts were labeled by a consensus of two independent investigators as previously described.^24^

### Model training and inference

**Figure 2.**
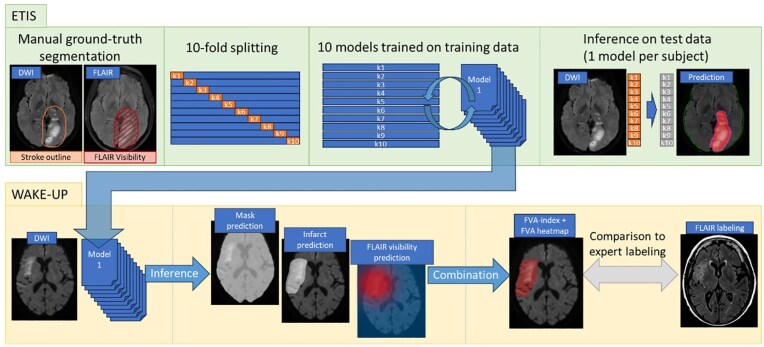
Illustration of study design.

The study design is illustrated in Figure 2. Preprocessing pipeline is described in Supplemental Material. Briefly, we performed a 10-fold cross-validation split^25^ onto the derivation cohort, with stratification on onset-to-imaging delay, MRI field strength and MR manufacturer. In each training fold, a DeepLabV3+ model^26^ (FVA-model) was trained to generate, using DWI sequence as input, 1/brain mask, 2/infarct region, and 3/FVA. Inference was performed in each validation fold. FVA-index was defined as the volume predicted as positive in the three prediction maps.

In the validation cohort, inference was performed using an ensemble model from the 10-fold cross-validation models trained on the derivation cohort. Brain mask and infarct regions were determined by soft majority voting^27^ across the 10 models. FVA probability was averaged across 10 predictions, resulting in a scalar value between 0 and 1 in each voxel. FVA-index was calculated as the total sum of these scalar values within infarct region. FVA prediction heatmaps were generated in a color scale ranging from red (FVA probability = 1) to blue (FVA probability = 0).

### Statistical analysis

Statistical analyses were performed with R (version 4.1; R Foundation). Relationship between FVA-index and DWI lesion volume was assessed with Pearson correlation coefficient. Dice score between ground-truth and predictions was computed in derivation cohort for infarct region segmentation, and for FVA segmentation in subjects without DWI-FLAIR mismatch. Receiver Operating Characteristic (ROC) and Area under ROC curve (AUC) evaluated the ability of low FVA-index values to predict visual DWI-FLAIR mismatch. An FVA-index cutoff was chosen using Youden Index, allowing to compute κ, sensitivity, specificity, accuracy, and predictive values. In the validation cohort, FVA-index’s diagnostic performances were compared to that of the “on-site assessment” with McNemar test.

Subgroup analyses were conducted in 1/lacunar versus non-lacunar infarcts, 2/<5 mL versus ⩾5 mL infarcts, 3/posterior fossa infarcts. The following post-hoc analyses were performed: 1/relation between onset-to-imaging delay and FVA-index, 2/performances in each cross-validation fold; 3/according to manufacturer and magnetic field strength; 4/according to onset-to-imaging delay; 5/ training on day-0 MRIs only; 6/training with reduced number of cases; 7/outcome analysis; 8/performances in excluded patients.

Values are expressed with mean ± standard deviation, or median, interquartile range (IQR). Distributions were compared with Mann-Whitney-*U* test. AUCs were compared with DeLong’s method. Statistical significance threshold was *p* < 0.05.

## Results

### Patients and MRI set characteristics

In the derivation cohort, 3980 datasets with paired DWI and FLAIR were screened. After exclusion of 375 with insufficient quality, 3605 MRI (2453 [68%] day-0 and 1152 [32%] day-1) in 2922 patients (1475 women, mean age: 70.5 years ± 15) were included.

In the validation cohort, 1343 patients were screened. After exclusion of datasets with technical issues (234), hemorrhages (70) and non-assessable mismatch (195), we included 844 day-0 MRIs of 844 patients (535 men, mean age: 65.5 years ± 11.4). Among them, 394 (46.7%) had been randomized in the WAKE-UP trial.

Detailed flowcharts are presented in Figure 1 and clinical data in Table 1. Cross-validation folds of the derivation cohort are detailed in Supplemental Table S1.

**Table 1.** Description of population in derivation and validation cohorts.

Population characteristic	Derivation cohort (ETIS)	Validation cohort (WAKE-UP)
Patients	2922	844
Women	1475 (50)	309 (37)
Age (years)	70.5 ± 15.0	65.6 ± 11.4
NIHSS	15 (9–20)	6 (4–10)
WAKE-UP randomization		
Yes	-	394 (47)
No	-	450 (53)
Treatment		
Intravenous thrombolysis	1500 (51)	205 (30)
Mechanical thrombectomy	2444 (84)	-
MRI	3605	844
MRI type		
Before treatment (day-0)	2453 (68)	844 (100)
Early follow-up (day-1)	1152 (32)	-
DWI lesion volume (mL)^a^	14.1 (4.4–40)	5.7 (1.5–18.2)
Manufacturer		
Siemens	2274 (63)	376 (45)
General Electric	809 (22)	178 (21)
Philips	522 (15)	290 (34)
MRI field strength		
1.5 Tesla	2452 (68)	492 (58)
3.0 Tesla	1153 (32)	352 (42)
Onset-to-imaging (h)^a^	2.2 (1.6–3.1)	-
<3h	1122 (46)	-
3–6 h	268 (11)	-
>6h	113 (4)	-
Unknown stroke onset	950 (39)	844 (100)

NIHSS: National Institutes of Health Stroke Scale.

Figures are given as number (percentage), mean ± standard deviation, or median (Inter Quartile Range, IQR), as appropriate.

^a^Onset-to-imaging and DWI lesion volume are given in the subgroup of day-0 MRIs.

### Derivation cohort

A visual “DWI-FLAIR mismatch” was present in 1457/3605 (40%) of all MRIs and 1440/2453 (58.7%) of day-0 MRIs. Median Dice score for infarct region segmentation was 0.76 (IQR 0.61–0.85). In the 2148/3605 (60%) MRIs without DWI-FLAIR mismatch, the Dice score for FVA segmentation was 0.68 [IQR 0.36–0.82].

The median FVA-index across all MRIs (*n* = 3605) was 6.6 (IQR 0–77.5) and was smaller on day-0 MRI (*n* = 2453, 0.02 [IQR 0–14.1]) compared to day-1 MRI (*n* = 1152, 100.5 [IQR 46.3–195.7]) (*p* < 0.001). On day-0 MRI, the median DWI lesion volume was 14.1 mL (IQR 4.4–40.0). The FVA-index correlated moderately with the DWI lesion volume (*r* = 0.30, *p* < 0.001).

**Figure 3.**
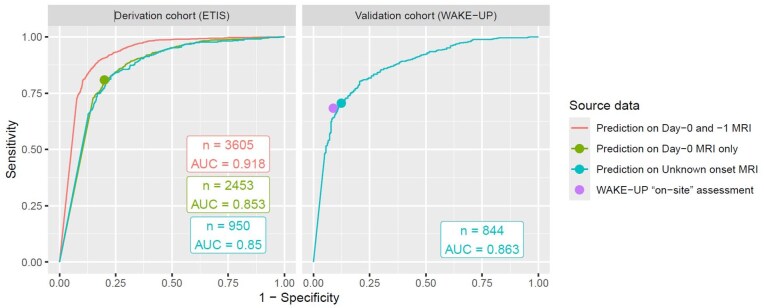
ROC curves for the prediction of DWI-FLAIR mismatch based on FVA-index. The optimal FVA-index cutoff was chosen using Youden Index on the day-0 MRI cohort (green dot in derivation cohort, and cyan dot in validation cohort). The sensitivity and specificity using the “on-site DWI-FLAIR mismatch assessment” in the WAKE-UP trial is shown as a purple dot.

The AUC for “DWI-FLAIR mismatch” prediction using FVA-index on day-0 MRI was 0.85 (95%CI: 0.84–0.87) and the derived optimal cutoff value was 0.5 (Figure 3). In patients with unknown onset-time (*n* = 950), the AUC was 0.85 (95%CI: 0.83–0.87).

The 0.5 FVA-index cutoff identified the visual “DWI-FLAIR mismatch” with a κ of 0.60 (95%CI: 0.57–0.63), 81% sensitivity (1164/1440; 95%CI: 79–83%) and 80% specificity (811/1013; 95%CI: 77–82%; Table 2).

**Table 2.** Performance of FVA-index for visual DWI-FLAIR mismatch identification in derivation and validation cohort.

Performance metric	Derivation cohort (*n* = 2453)	Validation cohort (*n* = 844)
Sensitivity	1165/1440 (81 [79–83])	378/537 (70 [66–74])
Specificity	811/1013 (80 [77–82])	269/307 (88 [83–91])
Predictive positive value	1165/1367 (85 [83–87])	378/416 (91 [88–93])
Negative predictive value	811/1086 (75 [72–77])	269/428 (63 [58–67])
Accuracy	1976/2453 (81 [79–82])	647/844 (77 [74–79])
κ	0.60 [0.57–0.63]	0.54 [0.48–0.59]

Diagnostic value was analyzed in day-0 MRI from the derivation cohort (*n* = 2453).

Values are expressed as numbers of subjects or κ values, with percentages in parentheses and 95% confidence intervals in brackets.

### Validation cohort

The visual “DWI-FLAIR mismatch,” based on final rating, was present in 537/844 (64%) and absent in 307/844 (36%). The median FVA-index was 0.6 (IQR 0–6.52) and the median DWI lesion volume was 5.7 mL (IQR 1.5–18.2 mL). The FVA-index correlated moderately with the DWI lesion volume (*r* = 0.47, *p* < 0.001).

**Figure 4.**
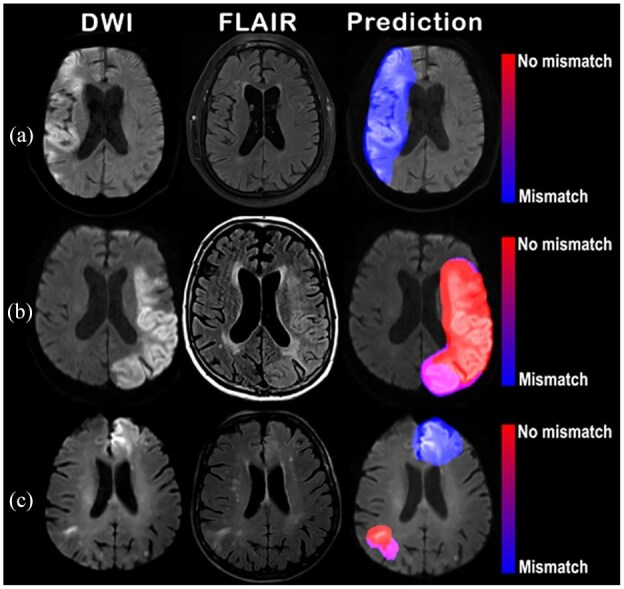
Examples of heatmaps generated by the FVA-model. Three illustrative cases. Left column shows *b* = 1000 s/mm^2^ DWI. Middle column shows the FLAIR sequence. Right column shows DWI with a transparent overlay of the FVA-map. In (a), a 67-year old woman presented a right middle cerebral artery infarct. FVA- predicted “DWI-FLAIR mismatch” (FVA-index = 0), matching the ground truth. In (b), a 76-year old woman presented a left middle cerebral artery infarct. The FVA-model predicted “no DWI-FLAIR mismatch” (FVA-index = 250), matching the ground truth. In (c), a 70-year old man presented with bilateral middle cerebral artery infarcts. The FVA-model predicted “no DWI-FLAIR mismatch” (FVA-index = 33), matching the ground truth. Heatmap showed “no DWI-FLAIR mismatch” on the right infarct, and “DWI-FLAIR mismatch” on the left infarct, matching the visual analysis of FLAIR, as the right infarct was clearly visible on FLAIR (arrow, middle column), whereas the left one was not.

The AUC for “DWI-FLAIR mismatch” prediction using FVA-index was 0.86 (95%CI: 0.84–0.89) with ROC curves presented in Figure 3. The 0.5 FVA-index cutoff identified the visual “DWI-FLAIR mismatch” with a κ of 0.54 (95%CI: 0.48–0.59), 70% sensitivity (378/537; 95%CI: 66–74%) and 88% specificity (269/307; 95%CI: 83–91%; Table 2). Illustrative heatmaps generated by the FVA-model are presented in Figure 4.

### Performance of “on-site assessment” versus FVA-model

The initial “on-site assessment” of the DWI-FLAIR mismatch in the WAKE-UP trial had the following performances for determining the final rating: κ = 0.56 (95%CI: 0.51–0.61), 68% sensitivity (367/537; 95%CI: 64–72%) and 91% specificity (280/307; 95%CI: 88–94%). These metrics did not differ from those obtained with the FVA-model for κ (0.56 vs 0.54, *p* = 0.83), sensitivity (68% vs 70%, *p* = 0.44) and specificity (91% vs 88%, *p* = 0.13).

### Subgroup analyses in validation cohort

Lacunar infarct were present in 178/844 (21%) MRIs. Median FVA-index was 0.2 (IQR 0–2.3) for lacunar versus 0.8 (IQR 0–8.7) for non-lacunar infarcts (*p* < 0.001). AUC for “DWI-FLAIR mismatch” prediction using FVA-index did not differ between lacunar and non-lacunar infarcts (0.83 [95%CI: 0.76–0.89] vs 0.87 [95%CI: 0.85–0.90], *p* = 0.20).

Volume infarct was <5 mL in 403/844 (47.7%) MRIs. AUC for “DWI-FLAIR mismatch” prediction using FVA-index did not differ between <5 and ⩾5 mL (0.85 [95%CI: 0.81–0.98] vs 0.88 [95%CI: 0.84–0.91], *p* = 0.35).

Posterior fossa infarcts were present in 65/844 (8%) MRIs, with 50/65 (77%) DWI-FLAIR mismatches. AUC for “DWI-FLAIR mismatch” prediction using FVA-index in posterior fossa infarcts was 0.88 [95%CI: 0.77–0.98].

### Post-hoc *analyses*

No performance difference was shown across different manufacturers and field strengths (Supplemental Table S2). Post-hoc analyses are detailed in Supplemental Material.

## Discussion

In this retrospective study, we developed a model for the assessment of the DWI-FLAIR mismatch based on DWI data only. We trained the FVA-model in a large expert-annotated database and validated its performance on external data from the WAKE-UP international trial, which demonstrated the benefit for thrombolysis in patients with unknown onset-time and DWI-FLAIR mismatch.^7^

FVA-model allowed to establish robust diagnostic metrics in the validation cohort. The methodological approach has the following strengths and novelty as compared to existing models ^11–17,28^: 1/training on data with expert-labeling DWI-FLAIR mismatch; 2/validation on a reference dataset from a clinical trial; 3/prediction based on input of DWI data only.

Comparing the performances of FVA-model with literature is challenging, as prior studies aimed to predict the onset-to-imaging delay, not the mismatch presence. In contrast, our aim was to develop a model for predicting the presence of DWI-FLAIR mismatch, relying on expert annotations as ground truth. Despite this distinction, FVA-model reached an AUC of 0.86, competing with AUC ranging from 0.63 to 0.90 in other studies.^11–17,28^ Although both DWI and FLAIR were required for the ground-truth visual assessment, we trained FVA-model solely based on DWI images and validated our findings in a large clinical trial cohort.

In the WAKE-UP trial, MRI for randomization was evaluated by a large group of investigators with various levels of expertise. The role for AI in medicine could be to assist clinicians, not necessarily outperform them. Therefore, it seems reasonable to aim for an accuracy similar to clinical practice using a Deep-Learning model. We showed similar performances for the FVA-index compared to the “on-site assessment” of WAKE-UP trial investigators (reflecting clinical practice). The consistent performances of FVA-model across derivation and validation cohorts, despite differences in ischemic lesion volumes, as well as in lacunar and non-lacunar infarcts and across varying MR manufacturers, underscores its generalizability and applicability in multiple clinical contexts.

We trained the FVA-model solely on DWI, given that DWI-FLAIR mismatch information is embedded within DWI data.^19–21,29^ This improves clinical utility by allowing off-line FVA computation immediately after DWI acquisition, thus accelerating the decision-making process. This model could be particularly useful in restless patients, since FLAIR is prone to motion artifacts, as evidenced by the 6% rate of low-quality FLAIR in the derivation cohort. Moreover, this approach allows to perform a prediction without prior coregistration in a context where head displacements are frequent between DWI and FLAIR, thereby improving algorithm speed and reducing the risk of partial volume effect.

The FVA-index produced by our model combines detected infarct area size with mismatch probabilities into a quantitative value. In addition to this scalar value, we think that the generated FVA heatmaps may enable a rapid identification and characterization of detected infarcts, and allow to detect multiple infarcts at different time points in a single patient. In order to identify the areas with FLAIR changes, previous authors have proposed to use gradient-based saliency maps,^15^ generated post-hoc after model training.^30^ However, such saliency maps lack trustworthiness and are not resistant to perturbations in the input images.^31^ In contrast, our approach allows to directly predict FVA heatmaps as a model output, providing more direct control for lesion detection and classification.

Our study presents several limitations. First, model performances were validated on the WAKE-UP dataset, which, while comprehensive, is not representative of current clinical practice, since it excluded patients for whom mechanical thrombectomy was planned.^7^ Although patients with large vessel occlusion were present in the WAKE-UP cohort, the DWI-FLAIR mismatch has not yet been evaluated for selecting patients for mechanical thrombectomy.^32^ Second, our ground truth FVA labeling was performed by a single radiologist. The methodology implied DWI labeling before FLAIR labeling, and may thus have increased the likelihood of identifying an FVA. Third, this was a retrospective study without prospective validation. Fourth, the algorithm displayed a similar performance between 1.5T and 3T, but we cannot exclude the possibility that the model reproduced a systematic interpretation bias as sensitivity to FLAIR changes may be different between 1.5 and 3T.^8^ Moreover, the compatibility of our algorithm with alternative DWI such as non-echo planar DWI and multishot EPI should be evaluated. Fifth, since our study included only early stroke cases, we were unable to assess the impact of T2 shine-through effects, which become prominent after day 3.^33^ Sixth, the FVA-model was evaluated as a stand-alone tool, and the FVA-index was specifically employed as a surrogate for FVA to quantitatively assess its performance. In order to validate the FVA-model as an assistance tool for human readers, we aim at validating the use of FVA heatmaps for treatment decision in unknown stroke onset time. To encourage future studies, the FVA-model and its training weights will be published as open-source.

In conclusion, our study validates FVA-model as a reliable tool for evaluating DWI-FLAIR mismatch in patients with unknown stroke onset-time, without having recourse to FLAIR image acquisition. The robust methodology and strong diagnostic metrics support FVA-model’s potential to aid treatment decision in AIS. Future research should focus on assessing the added value of FVA heatmaps in prospective studies, as a support tool to increase diagnostic confidence.

## Supplementary Material

sj-docx-1-eso_23969873251362712

## Data Availability

The image data that support the findings of this study are available from ETIS registry and WAKE-UP Stroke trial, but restrictions apply to the availability of these data. Derivative data are however available from the authors upon reasonable request and with permission of ETIS registry and WAKE-UP Stroke trial scientific boards. Source code and model weights will be made publicly available on https://github.com/NeuroSainteAnne/FlairVisibilityArea.

## ORCID iDs

Joseph Benzakoun   https://orcid.org/0000-0002-0733-0104

Lauranne Scheldeman   https://orcid.org/0000-0002-5263-3550

Anke Wouters   https://orcid.org/0000-0001-5229-2699

Jens Fiehler   https://orcid.org/0000-0001-8533-7478

Ivana Galinovic   https://orcid.org/0000-0003-4904-8251

Keith W Muir   https://orcid.org/0000-0001-9535-022X

Salvador Pedraza   https://orcid.org/0000-0003-2517-4413

Josep Puig   https://orcid.org/0000-0003-2791-6599

Claus Z Simonsen   https://orcid.org/0000-0003-1363-0266

Vincent Thijs   https://orcid.org/0000-0002-6614-8417

Bailiang Chen   https://orcid.org/0000-0003-1038-7846

Grégoire Boulouis   https://orcid.org/0000-0001-8422-9205

Guillaume Turc   https://orcid.org/0000-0001-5059-4095
