# Supplementary material for: Automated DWI-FLAIR mismatch assessment in stroke using DWI only
Source: Eur Stroke J. 2026 Jan 1;11(1):23969873251362712. doi: 10.1093/esj/23969873251362712 (PMC12866262; doi:10.1093/esj/23969873251362712)
Supplement: sj-docx-1-eso_23969873251362712 [file sj-docx-1-eso_23969873251362712.docx]

**Supplemental Material for “Automated DWI-FLAIR Mismatch Assessment in Acute Stroke Using DWI only”**

[Supplemental methods 2](#_Toc201596636)

[1) Data type and resolution 2](#_Toc201596637)

[2) Preprocessing 2](#_Toc201596638)

[3) Deep learning model 3](#_Toc201596639)

[Supplemental Table S1: Population detail for each cross-validation fold of the derivation cohort 6](#_Toc201596640)

[Supplemental results 8](#_Toc201596641)

[Relation between FVA-index and Onset-to-imaging delay 8](#_Toc201596642)

[Supplemental Figure S1: Correlation between prediction and onset-to-imaging delay in derivation cohort 8](#_Toc201596643)

[Performances across cross-validation folds 9](#_Toc201596644)

[Supplemental Table S2: AUC for the prediction of DWI-FLAIR mismatch based on FVA-index in the derivation cohort, detailed for each cross-validation fold. 9](#_Toc201596645)

[Performances across manufacturers and field strength 10](#_Toc201596646)

[Supplemental Table S3: AUC for the prediction of DWI-FLAIR mismatch based on FVA-index, detailed for each manufacturer and field strength 10](#_Toc201596647)

[Ablation study (reduced number of training subjects) 11](#_Toc201596648)

[Supplemental Table S4: AUC for the prediction of DWI-FLAIR mismatch in ablation analysis 11](#_Toc201596649)

[Ablation study (Day-0 vs. Day-0 + Day-1) 12](#_Toc201596650)

[Supplemental Table S5: Comparison of main analysis and ablation study on principal metrics 12](#_Toc201596651)

[Model with precise infarct segmentations 13](#_Toc201596652)

[Supplemental Table S6: Comparison of main analysis and study with FVA defined using a precise infarct segmentation 13](#_Toc201596653)

[Outcome analysis 14](#_Toc201596654)

[Supplemental Table S7: Outcome analysis in different subgroup of randomized patients 14](#_Toc201596655)

[Excluded subjects analysis 15](#_Toc201596656)

[1) Subjects excluded from derivation cohort 15](#_Toc201596657)

[Supplemental Figure S2: Effect of sequence quality on model performances in the derivation cohort 15](#_Toc201596658)

[2) Subjects excluded from validation cohort 16](#_Toc201596659)

[Supplemental Figure S3: Example of model prediction on excluded patients 17](#_Toc201596660)

[Software deployment and performance 18](#_Toc201596661)

[Supplemental references 19](#_Toc201596662)

# Supplemental methods

### Data type and resolution

DWI sequences were EPI scans with 3 diffusion directions. The b=0 weighting, and the trace image for b=1000/mm² weighting were extracted. These weightings were chosen for their large retrospective availability across different centers. There was no standardization of the sequences across different centers. In derivation cohort, the mean ± SD in-plane resolution was 1.02mm ± 0.33, and the mean slice thickness 4.2mm ± 1.33. In validation cohort, the mean in-plane resolution was 0.91mm ± 0.04, and the mean slice thickness of 5.26mm ± 1.08.

### Preprocessing

MRI sets were pre-processed following previously published pipeline^1^.

#### DWI b=1000 – b=0 and DWI-FLAIR co-registration:

In cases where the two weightings of the DWI sequence were not properly aligned, a 6-parameter rigid co-registration of the b=0 weighting was performed onto the b=1000s/mm² weighting, using Advanced Normalization Tools version 2.3.5 (<https://stnava.github.io/ANTs>). The same procedure was applied in cases when FLAIR and DWI were not aligned.

#### Quality assessment

For each DWI and FLAIR sequence, a single reader assessed quality on an ordinal scale ranging from 1 to 3. For DWI, the following definition was used: 1/ non-diagnostic quality DWI; 2/ intermediate quality DWI impaired by some artifacts but clearly visible ischemic stroke; 3/ diagnostic quality DWI. For FLAIR, the following definition was used: 1/ non-diagnostic quality FLAIR; 2/ intermediate quality FLAIR impaired by some artifacts but assessable signal changes in the area of the ischemic stroke; 3/ diagnostic quality FLAIR.

#### Brain mask extraction

For each subject, a binary brain mask was computed through an Otsu method from both b=0 and b=1000 s/mm² images using the DIPY library^2^. Brain segmentation was manually adjusted after visual inspection.

#### Stroke segmentation

All acute ischemic stroke were roughly segmented with a 3D Region Of Interest (ROI) manually drawn using Mango software (version 4.0.1, Research Imaging Institute, San Antonio, Texas, non-commercial software). These loose ROIs were intersected with the previously computed brain mask to determine the “infarct region”.

#### FLAIR visibility ROIs

All stroke ROIs were duplicated onto the corresponding FLAIR sequence. The ROIs were then corrected manually in order to assess FLAIR visibility, such as only FLAIR visible areas would be kept in the FLAIR visibility ROIs.

#### Data harmonization

The ETIS and WAKE-UP-image database included many different MRI image matrix sizes. All MRI sets were either up-scaled or down-scaled into a standard 256×256 squared matrix size.

A Z-score normalization was performed to standardize the signal intensities of each DWI weightings by mean centering and variance scaling.

#### Data augmentation

Data augmentation was performed to artificially increase the database and prevent overfitting. It included: (1) flipping volume along x- and z-axis, (2) rotating image between -8 and +8 degrees, (3) shearing image between -8 and +8 degrees, (4) translating image between -4% and +4% along x-axis and y-axis, (5) scaling the image of a factor between 0.9 and 1.1, and (6) adjusting the image contrast and brightness between -30% and +30%.

#### Quality control

For each of the aforementioned pre-processing steps, we performed a systematic qualitative visual inspection to detect data pre-processing failure. Consequently, all image pre-processing steps were achieved and checked before feeding them into the network.

### Deep learning model

#### Architecture

Models were all developed on pytorch v2.2.0. The Deep-Learning model was created using SegmentationModelsPytorch^3^ and composed of:

- A DeepLabV3+ architecture^4^. This network combines Atrous Spatial Pyramid Pooling from DeepLabv1 and Encoder-Decoder Architecture from DeepLabv2 and is used for multiple image segmentation tasks
- An EfficientNet-b0^5^ encoder backbone. This encoder uniformly scales all dimensions of depth/width/resolution using a compound coefficient and achieves state-of-the art accuracy in many classification challenges. It was pretrained on imagenet data

#### Input

We wanted our model to take advantage of the 3D structure of stroke information. However, training and inference on 3D convolutional models is resource and time intensive and may not be adapted here because of the anisotropic nature of the DWI sequence along z-axis. As a result, we used an intermediate “2.5D” solution where each slice is predicted individually but based on a 7-slices thickness input. To this end, we performed a modification of the Segmentation Model Pytorch 3D module^6^ with customized strides along the z-axis. As a result, for the prediction of 1 individual slice, the input was composed of:

- - DWI b=0 s/mm² weighting (predicted slice + 3 slices above and 3 slices below)
  - DWI b=1000 s/mm² weighting (predicted slice + 3 slices above and below)

As in a previous study^7^ where the ADC map did not have added value for the prediction of FLAIR hyperintensities, we did not include the ADC map in the input. For extreme slices at the top and bottom of the DWI volume, zero-padding was applied, i.e. missing slices were filled with zeros to maintain a consistent input size.

#### Output

The model had three 256×256 maps as output:

- A binary mask of the brain
- A binary mask representing the rough stroke segmentation
- A binary mask representing the FLAIR visible areas.

#### Losses

- Three training losses corresponded to each output, and were computed as the binary cross-entropy loss between real output and predicted output, with a smoothing factor of 0.01. The FLAIR visibility loss was weighted by a factor of 100.
- The Stroke segmentation loss was masked so that training occurs only inside the brain mask. The FLAIR visibility loss was masked so that training occurs only inside the stroke mask

#### Training procedure

We performed a 10-fold cross-validation split onto the derivation cohort. The splitting procedure implied a stratified cross-validation^8^ with stratification on: 1/onset-to-imaging delay (<3-hrs, 3-6-hrs, >6-hrs or unknown), 2/MRI field strength (1.5 or 3 Tesla) and 3/MR manufacturer. As a result, each fold was trained on ≈2700 cases and validated on ≈300 cases with similar variable repartition (details given in Supplemental Table S1). When one patient had both day-0 and day-1 MRI, both were randomized into the same fold.

Before training, each training fold was further split into an inner training set (80%) and an inner validation set (20%), using the same stratification as the outer cross-validation procedure. Each model was trained onto the training data (80%) and monitored on the internal validation set (20%).

Detailed patient characteristics in each fold are presented in Supplemental Table S1.

The model was then trained during 75 epochs. Each epoch, the model was trained on 16384 slices randomly chosen from the training set, with data augmentation, in batches of 128 slices.

At the end of each epoch, inference was made on the inner validation set (without data augmentation) and patient-wise validation AUC and validation loss were monitored. The learning rate was set at 0.001 with an Adam optimizer. Learning rate was reduced using a scheduler (Plateau scheduler with 6 epochs patience, multiplicative factor at 0.1, and minimum learning rate set at 0.000001). The training process was stopped early when the validation AUC plateaued during more than 15 epochs. Training was performed onto a GPU server with 4 NVIDIA GeForce GTX 1080 Ti GPUs with 11GB VRAM each, 2 Intel Xeon Processor E5 CPU, 256GB RAM, using Ubuntu 20.04.

| Cross-validation Fold | All folds | 1 | 2 | 3 | 4 | 5 | 6 | 7 | 8 | 9 | 10 |
| --- | --- | --- | --- | --- | --- | --- | --- | --- | --- | --- | --- |
| Patients (n=) | 2922 | 293 | 293 | 292 | 292 | 292 | 292 | 292 | 292 | 292 | 292 |
| Women | 1475 (50.5%) | 149 (50.9%) | 165 (56.3%) | 147 (50.3%) | 139 (47.6%) | 153 (52.4%) | 147 (50.3%) | 128 (43.8%) | 139 (47.6%) | 155 (53.1%) | 153 (52.4%) |
| Age (years) | 70.5 (± 15) | 69.2 (± 15.7) | 70.2 (± 16.4) | 70.6 (± 14.8) | 72.2 (± 14.7) | 70.9 (± 14.9) | 70.8 (± 14.6) | 69.5 (± 14.7) | 70.8 (± 14.4) | 70.7 (± 14.3) | 70.2 (± 15.6) |
| NIHSS | 15 (IQR 9-20) | 15 (IQR 9-20) | 15 (IQR 9-19) | 15 (IQR 9-19) | 16 (IQR 10-20) | 14 (IQR 8-19) | 15 (IQR 9-19) | 14 (IQR 9-19) | 15 (IQR 9-20) | 15 (IQR 9-20) | 15 (IQR 9-20) |
| ASPECTS | 8 (IQR 6-9) | 8 (IQR 6-9) | 8 (IQR 6-9) | 8 (IQR 6-9) | 8 (IQR 6-9) | 8 (IQR 7-9) | 8 (IQR 6-9) | 8 (IQR 6-9) | 8 (IQR 6-9) | 8 (IQR 6-9) | 8 (IQR 6-9) |
| Treatment  - Intravenous Thrombolysis  - Mechanical Thrombectomy | 1500 (51.3%)  2444 (83.6%) | 145 (49.5%)  247 (84.3%) | 152 (51.9%)  255 (87%) | 160 (54.8%)  242 (82.9%) | 168 (57.5%)  251 (86%) | 139 (47.6%)  240 (82.2%) | 157 (53.8%)  233 (79.8%) | 145 (49.7%)  243 (83.2%) | 153 (52.4%)  246 (84.2%) | 144 (49.3%)  244 (83.6%) | 137 (46.9%)  243 (83.2%) |
| Stroke etiology  - Cardioembolic  - Atherosclerosis  - Dissection  - Other / cryptogenic | 1132 (38.7%)  419 (14.3%)  106 (3.6%)  1265 (43.3%) | 118 (40.3%)  51 (17.4%)  15 (5.1%)  109 (37.2%) | 122 (41.6%)  42 (14.3%)  8 (2.7%)  121 (41.3%) | 116 (39.7%)  37 (12.7%)  19 (6.5%)  120 (41.1%) | 124 (42.5%)  33 (11.3%)  8 (2.7%)  127 (43.5%) | 102 (34.9%)  46 (15.8%)  12 (4.1%)  132 (45.2%) | 106 (36.3%)  39 (13.4%)  13 (4.5%)  134 (45.9%) | 106 (36.3%)  35 (12%)  10 (3.4%)  141 (48.3%) | 105 (36%)  52 (17.8%)  7 (2.4%)  128 (43.8%) | 114 (39%)  48 (16.4%)  8 (2.7%)  122 (41.8%) | 119 (40.8%)  36 (12.3%)  6 (2.1%)  131 (44.9%) |
| MRI (N=) | 3605 | 362 | 362 | 360 | 360 | 360 | 363 | 362 | 359 | 359 | 358 |
| MRI type  - Before treatment (baseline)  - Early follow-up | 2453 (68%)  1152 (32%) | 246 (68%)  116 (32%) | 246 (68%)  116 (32%) | 244 (67.8%)  116 (32.2%) | 244 (67.8%)  116 (32.2%) | 245 (68.1%)  115 (31.9%) | 246 (67.8%)  117 (32.2%) | 245 (67.7%)  117 (32.3%) | 246 (68.5%)  113 (31.5%) | 246 (68.5%)  113 (31.5%) | 245 (68.4%)  113 (31.6%) |
| DWI lesion volume (mL)* | 14.1 (IQR 4.4-40) | 18.2 (IQR 4.8-39.6) | 14.7 (IQR 4.2-41.3) | 15.4 (IQR 3.6-40.4) | 13.1 (IQR 4.2-45.2) | 10.9 (IQR 3.6-33.3) | 13.7 (IQR 4.9-43.6) | 13 (IQR 3.7-32.8) | 14.4 (IQR 5.2-36.7) | 18.1 (IQR 5.1-44.7) | 14.3 (IQR 5.4-42.1) |
| Manufacturer  - Siemens  - GE  - Philips | 2274 (63.1%)  809 (22.4%)  522 (14.5%) | 230 (63.5%)  78 (21.5%)  54 (14.9%) | 228 (63%)  79 (21.8%)  55 (15.2%) | 231 (64.2%)  77 (21.4%)  52 (14.4%) | 229 (63.6%)  80 (22.2%)  51 (14.2%) | 226 (62.8%)  83 (23.1%)  51 (14.2%) | 231 (63.6%)  82 (22.6%)  50 (13.8%) | 229 (63.3%)  82 (22.7%)  51 (14.1%) | 223 (62.1%)  84 (23.4%)  52 (14.5%) | 228 (63.5%)  81 (22.6%)  50 (13.9%) | 219 (61.2%)  83 (23.2%)  56 (15.6%) |
| MRI Field strength  - 1.5 Tesla  - 3.0 Tesla | 2452 (68%)  1153 (32%) | 245 (67.7%)  117 (32.3%) | 240 (66.3%)  122 (33.7%) | 244 (67.8%)  116 (32.2%) | 246 (68.3%)  114 (31.7%) | 248 (68.9%)  112 (31.1%) | 254 (70%)  109 (30%) | 252 (69.6%)  110 (30.4%) | 249 (69.4%)  110 (30.6%) | 237 (66%)  122 (34%) | 237 (66.2%)  121 (33.8%) |
| Onset-to-imaging delay (h) * in day-0 MRI  - < 3h  - 3-6h  - > 6  - Unknown stroke onset | 2.2 (IQR 1.6-3.1)  1122 (45.7%)  268 (10.9%)  113 (4.6%)  950 (38.7%) | 2.1 (IQR 1.7-3.1)  113 (45.9%)  26 (10.6%)  12 (4.9%)  95 (38.6%) | 2.2 (IQR 1.6-3.2)  114 (46.3%)  26 (10.6%)  11 (4.5%)  95 (38.6%) | 2.1 (IQR 1.7-3.1)  113 (46.3%)  25 (10.2%)  12 (4.9%)  94 (38.5%) | 2.2 (IQR 1.6-2.8)  115 (47.1%)  24 (9.8%)  9 (3.7%)  96 (39.3%) | 2.3 (IQR 1.7-3)  109 (44.5%)  28 (11.4%)  10 (4.1%)  98 (40%) | 2.2 (IQR 1.6-3.1)  109 (44.3%)  29 (11.8%)  11 (4.5%)  97 (39.4%) | 2.2 (IQR 1.6-3.3)  109 (44.5%)  30 (12.2%)  11 (4.5%)  95 (38.8%) | 2.2 (IQR 1.7-3.2)  113 (45.9%)  28 (11.4%)  12 (4.9%)  93 (37.8%) | 2.1 (IQR 1.6-3.2)  114 (46.3%)  27 (11%)  12 (4.9%)  93 (37.8%) | 2.2 (IQR 1.7-3.1)  113 (46.1%)  25 (10.2%)  13 (5.3%)  94 (38.4%) |

### Supplemental Table S1: Population detail for each cross-validation fold of the derivation cohort

* DWI lesion volume and Onset-to-imaging delay are given in the subgroup on Baseline MRIs

#### Inference

For final inference in derivation and validation cohorts, the predicted output maps were first binarized (using a fixed threshold at 0). Then, an intersection was performed between stroke prediction and brain mask to define the “infarct region”; similarly, an intersection was performed between FLAIR visibility and infarct region to define the final FLAIR Visibility Area (FVA).

In the derivation cohort, the FVA-index was defined as the number of positive voxels within the FVA, multiplied by voxel volume.

In the validation cohort, inference was performed using an ensemble model from the 10-fold cross-validation models trained on the derivation cohort. Brain mask and infarct regions were determined by soft majority voting across the 10 models. This majority voting was performed for defining a unique infarct region for each patient. FVA probability was averaged across 10 predictions, resulting in a scalar value between 0 and 1 in each voxel. FVA-index was calculated as the total sum of these scalar values within infarct region.

#### Data visualisation

All predictions were exported as RGB NIFTI format as an overlay onto the DWI sequence, color scale ranging from red (FVA probability = 1) to blue (FVA probability = 0).

# Supplemental results

## Relation between FVA-index and Onset-to-imaging delay

In day-0 MRIs from patients with known onset-time in the derivation cohort (n=1503), using the 0.5 FVA-index cutoff, the predicted percentage of patients without “DWI-FLAIR Mismatch” increased with onset-to-imaging delay: 65/299 (22%) before 1.5h, 261/834 (31%) between 1.5h and 3h, 84/184 (46%) between 3h and 4.5h, 53/77 (69%) between 4.5h and 6h, 48/62 (77%) between 6h and 9h, and 39/47 (83%) after 9h (Supplemental Figure S1).

### Supplemental Figure S1: Correlation between prediction and onset-to-imaging delay in derivation cohort


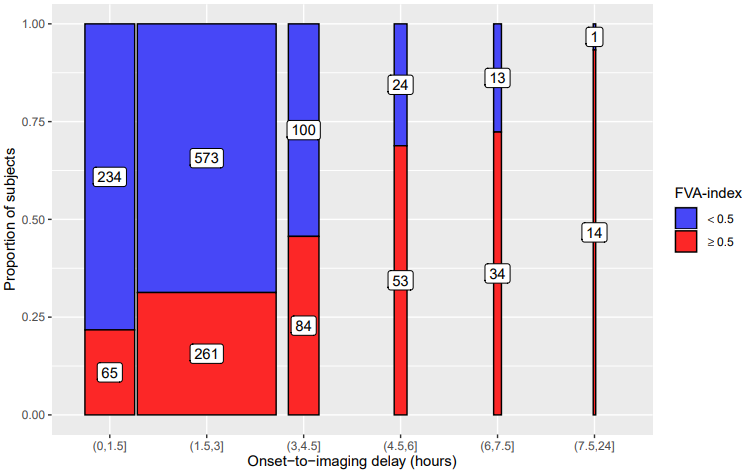


The number of subjects is overlayed onto each bar. Bar area is proportional to the number of subjects in each category.

## Performances across cross-validation folds

### Supplemental Table S2: AUC for the prediction of DWI-FLAIR mismatch based on FVA-index in the derivation cohort, detailed for each cross-validation fold, and in the subgroup of day-0 MRI only.

| K-Fold | Number of MRI (inner training subset) | Number of MRI (inner validation subset) | AUC | Number of MRI (inner validation subset, day-0 MRI) | AUC (day-0 MRI) |
| --- | --- | --- | --- | --- | --- |
| 1 | 3243 | 362 | 0.910 [0.875-0.945] | 246 | 0.842 [0.793-0.890] |
| 2 | 3243 | 362 | 0.904 [0.868-0.940] | 246 | 0.840 [0.791-0.889] |
| 3 | 3245 | 360 | 0.919 [0.888-0.951] | 244 | 0.841 [0.792-0.889] |
| 4 | 3245 | 360 | 0.919 [0.887-0.951] | 244 | 0.849 [0.802-0.896] |
| 5 | 3245 | 360 | 0.920 [0.888-0.952] | 245 | 0.858 [0.813-0.904] |
| 6 | 3242 | 363 | 0.931 [0.900-0.961] | 246 | 0.875 [0.832-0.918] |
| 7 | 3243 | 362 | 0.920 [0.888-0.952] | 245 | 0.854 [0.807-0.900] |
| 8 | 3246 | 359 | 0.910 [0.876-0.944] | 246 | 0.856 [0.81-0.902] |
| 9 | 3246 | 359 | 0.900 [0.865-0.935] | 246 | 0.819 [0.767-0.87] |
| 10 | 3247 | 358 | 0.947 [0.920-0.973] | 245 | 0.902 [0.864-0.94] |

## Performances across manufacturers and field strength

In the 6 subgroups composed from each 3 Manufacturers and 2 Magnetic field strengths, AUC for DWI-FLAIR mismatch determination using FVA-index were 0.82 to 0.87 in derivation cohort and 0.82 to 0.91 in validation cohort (detailed results in Supplemental Table S2). Pairwise AUC comparisons did not show significant difference (P>0.54).

|  | Derivation cohort (ETIS) | | Validation cohort (WAKE-UP) | |
| --- | --- | --- | --- | --- |
|  | n | AUC (95%CI) | n | AUC (95%CI) |
| Siemens 3T | 362 | 0.830 (0.783-0.877) | 125 | 0.909 (0.849-0.969) |
| Siemens 1.5T | 997 | 0.875 (0.852-0.898) | 251 | 0.864 (0.815-0.914) |
| General Electric 3T | 169 | 0.823 (0.753-0.894) | 29 | 0.857 (0.681-1) |
| General Electric 1.5T | 474 | 0.828 (0.788-0.868) | 149 | 0.867 (0.795-0.939) |
| Philips 3T | 275 | 0.844 (0.793-0.895) | 198 | 0.857 (0.798-0.917) |
| Philips 1.5T | 176 | 0.861 (0.803-0.919) | 92 | 0.828 (0.736-0.920) |

### Supplemental Table S3: AUC for the prediction of DWI-FLAIR mismatch based on FVA-index, detailed for each manufacturer and field strength

Pairwise comparison between subgroups did not show any statistical difference:

- Derivation cohort: Siemens 1.5T vs. General Electric 1.5T: *P*=0.54
- All other comparisons: *P*>0.99

## Ablation study (reduced number of training subjects)

In order to evaluate the minimum number of subjects needed for accurate training, we conducted an ablation study on the first cross-validation fold of our derivation cohort. To this effect, we retrained the first cross-validation fold after adjusting the size of the inner test set. The number of MRI in the inner validation set and outer validation set remained constant (respectively, 649 and 362).

In the chosen fold, the validation AUC remained stable even when the training set was reduced to as few as 500 MRI scans. Using the same methodology (20% inner validation set and outer cross-validation with 10 folds), the minimum number of subjects per cross-validation fold would then be 625, and the total number of subjects 688. Factoring in an additional margin for error, the approximate minimal number of cases necessary for effective FVA training should be on the order of 1000 subjects.

| Number of subjects in inner test set | AUC in Inner validation set (n=649) | AUC in Outer validation set (n=362) | P-value | AUC in Outer validation set, day-0 only (n=246) |
| --- | --- | --- | --- | --- |
| Reference (full set) 2594 | 0.917 | 0.910 [95%CI: 0.880 - 0.91] | (ref) | 0.842 [95%CI: 0.791 - 0.842] |
| 2500 | 0.912 | 0.921 [95%CI: 0.893 - 0.921] | 0.12 | 0.870 [95%CI: 0.826 - 0.870] |
| 2000 | 0.913 | 0.912 [95%CI: 0.883 - 0.912] | 0.79 | 0.851 [95%CI: 0.803 - 0.851] |
| 1500 | 0.908 | 0.903 [95%CI: 0.872 - 0.903] | 0.39 | 0.835 [95%CI: 0.784 - 0.835] |
| 1000 | 0.911 | 0.906 [95%CI: 0.876 - 0.906] | 0.66 | 0.843 [95%CI: 0.794 - 0.843] |
| 750 | 0.913 | 0.921 [95%CI: 0.893 - 0.921] | 0.27 | 0.865 [95%CI: 0.820 - 0.865] |
| 500 | 0.878 | 0.899 [95%CI: 0.867 - 0.899] | 0.30 | 0.825 [95%CI: 0.773 - 0.825] |
| 250 | 0.890 | 0.889 [95%CI: 0.855 - 0.889] | 0.055 | 0.812 [95%CI: 0.757 - 0.812] |
| 100 | 0.776 | 0.811 [95%CI: 0.765 - 0.811] | <0.0001 | 0.730 [95%CI: 0.667 - 0.730] |

### Supplemental Table S4: AUC for the prediction of DWI-FLAIR mismatch in ablation analysis

## Ablation study (Day-0 vs. Day-0 + Day-1)

We initially hypothesized that including early follow-up MRI at day-1 should improve the training process. Indeed, including stroke at later stage should increase data diversity, and compensate for the fact that MRI performed beyond 6 hours are rare in the ETIS dataset (165/2435 day-0 MRI, 6,8%) due to its inclusion criteria (patients referred for mechanical thrombectomy). In order to test this hypothesis, we performed an ablation analysis. We re-trained completely the deep-learning models after restricting the input data to select only day-0 MRIs. Then, we performed the same analyses as in our main analysis and compared the paired results between main study and ablation study. The detailed results are presented in Supplemental Table S3. After removing day-1 MRI from the training data, model performances were globally reduced. AUC for DWI-FLAIR mismatch determination using FVA-index in the validation cohort was significantly decreased as compared to main analysis (0.826 vs. 0.863, *P*<0.001).

|  | Training on  Day-0 and Day-1 (main analysis) | Training on  Day-0 only (ablation study) | *P* value |
| --- | --- | --- | --- |
| **Derivation cohort (ETIS): n=3605** |  |  |  |
| AUC for DWI-FLAIR mismatch prediction using FVA-index in all MRI (n=3605) | 0.92 (0.91-0.93) | 0.91 (0.90-0.91) | <0.001 |
| AUC for DWI-FLAIR mismatch prediction using FVA-index in D0 MRI (n=2453) | 0.85 (0.84-0.87) | 0.85 (0.83-0.87) | 0.57 |
| AUC for DWI-FLAIR mismatch prediction using FVA-index in unknown-onset D0 MRI (n=950) | 0.85 (0.83-0.87) | 0.84 (0.81-0.86) | 0.14 |
| Best FVA-index threshold derived in D0 MRI | 0.5 | 4.8 | - |
| **Validation cohort (WAKE-UP): n=844** |  |  |  |
| AUC for DWI-FLAIR mismatch prediction using FVA-index | 0.86 (0.84-0.89) | 0.83 (0.80-0.85) | <0.001 |
| Kappa between thresholded FVA-index and  DWI-FLAIR mismatch labeling | 0.54 (0.48-0.59) | 0.46 (0.40-0.51) | 0.61 |
| Sensitivity of thresholded FVA-index for DWI-FLAIR mismatch prediction | 70% (66-74%) | 67% (63-71%) | 0.049 |
| Specificity of thresholded FVA-index for DWI-FLAIR mismatch prediction | 88% (83-91%) | 82% (78-87%) | 0.009 |

### Supplemental Table S5: Comparison of main analysis and ablation study on principal metrics

## Model with precise infarct segmentations

One of our hypotheses was that using large, loosely-defined stroke regions would increase our sensitivity to the detection of stroke, by taking into account regionalized prediction. In order to test this hypothesis, we evaluated the model performance after redefining the FVA as the intersection of original FVA with a more precise stroke segmentation performed using ADSv1.3.^9^ The detailed results are presented in Supplemental Table S4. After defining FVA using a precise infarct segmentation rather than a loosely-defined ROI, model performances were globally reduced. AUC for DWI-FLAIR mismatch determination using FVA-index in the validation cohort was significantly decreased as compared to main analysis (0.825 vs. 0.863, *P*<0.001). There was a tendency towards reduced sensitivity using the precise segmentation (68% vs. 70%, *P*=0.07).

|  | FVA defined using stroke region (original model) | FVA defined using precise infarct segmentation | *P* value |
| --- | --- | --- | --- |
| **Derivation cohort (ETIS): n=3605** |  |  |  |
| AUC for DWI-FLAIR mismatch prediction using FVA-index in all MRI (n=3605) | 0.92 (0.91-0.93) | 0.88 (0.87-0.89) | <0.001 |
| AUC for DWI-FLAIR mismatch prediction using FVA-index in D0 MRI (n=2453) | 0.85 (0.84-0.87) | 0.80 (0.79-0.82) | <0.001 |
| AUC for DWI-FLAIR mismatch prediction using FVA-index in unknown-onset D0 MRI (n=950) | 0.85 (0.83-0.87) | 0.81 (0.79-0.84) | <0.001 |
| Best FVA-index threshold derived in D0 MRI | 0.5 | 0.004 | - |
| **Validation cohort (WAKE-UP): n=844** |  |  |  |
| AUC for DWI-FLAIR mismatch prediction using FVA-index | 0.86 (0.84-0.89) | 0.83 (0.80-0.85) | <0.001 |
| Kappa between thresholded FVA-index and  DWI-FLAIR mismatch labeling | 0.54 (0.48-0.59) | 0.49 (0.43-0.54) | 0.74 |
| Sensitivity of thresholded FVA-index for DWI-FLAIR mismatch prediction | 70% (66-74%) | 68% (64-72%) | 0.07 |
| Specificity of thresholded FVA-index for DWI-FLAIR mismatch prediction | 88% (83-91%) | 85% (81-89%) | 0.20 |

### Supplemental Table S6: Comparison of main analysis and study with FVA defined using a precise infarct segmentation

## Outcome analysis

In patients randomized in the WAKE-up trial, the 3-month modified Rankin Scale (mRS) was collected as the primary endpoint of the trial. In these subjects, we performed a shift analysis in order to evaluate the effect of rtPA treatment as compared to placebo on the 3-month mRS (i.e., improving outcome).

This shift analysis was performed using an ordered logistic regression (using package “MASS” from R) after checking the parallel regression assumption using the Brant test. The logistic regression was adjusted on the same parameters as the WAKE-UP trial study, i.e., age and NIHSS at admission. The shift analysis was repeated in the following subgroups: subjects with DWI-FLAIR mismatch according to CIRB (i.e., per-protocol analysis), and in the subgroup of subjects with FVA-index <0.5 and ≥0.5. We also performed an interaction analysis between randomization group (rtPA vs. placebo) and FVA-index (<0.5 vs. ≥0.5).

Detailed results are shown in Supplemental Table S7. We reproduced the conclusions of the WAKE-UP study in our subset, as the rtPA was associated with better outcomes (adjusted OR = 0.63 [0.44-0.92]).

In the subgroup of patients with low FVA-index (<0.5) (n=270), the effect had a tendency to be stronger than in the whole group (adjusted OR = 0.57 [0.37-0.88]). In the subgroup of patients with higher FVA-indices (≥ 0.5) the effect of rtPA on outcome was not statistically significant (P=0.714), potentially due to the lack of statistical power (n=116). We did not observe an interaction of the FVA-index on the treatment effect of tPA on outcome (P=0.26).

|  | Total N= | N in rtPA group | N in placebo group | Adjusted Odds Ratio | Adjusted P-value |
| --- | --- | --- | --- | --- | --- |
| All randomized subjects included in our study | 386 | 200 | 186 | 0.63 [0.44-0.92] | 0.016 |
| Subgroup of randomized subjects with DWI-FLAIR mismatch according to CIRB | 360 | 184 | 176 | 0.65 [0.44-0.95] | 0.025 |
| Subgroup of randomized subjects with FVA-index < 0.5 | 270 | 140 | 130 | 0.57 [0.37-0.88] | 0.011 |
| Subgroup of randomized subjects with FVA-index ≥ 0.5 | 116 | 60 | 56 | 0.87 [0.45-1.72] | 0.71 |

### Supplemental Table S7: Outcome analysis in different subgroup of randomized patients

## Excluded subjects analysis

This sensitivity analysis was performed to evaluate how the model performed outside of its initial specifications. Inference was performed in subjects excluded both from derivation and validation cohort, whenever DWI sequence could be processed.

### Subjects excluded from derivation cohort

In the derivation cohort, causes for exclusion were insufficient FLAIR and/or DWI quality. The “insufficient quality” labeling was further divided in “low” and “intermediate” qualities.

AUC for DWI-FLAIR mismatch prediction based on FVA-index in the 420 excluded patients is summarized in Supplemental Figure S2. Interestingly, AUC was preserved in the subgroup with intermediate DWI and FLAIR quality (n=90, AUC=0.93, 95%CI: 0.87-0.99). This AUC was significantly higher than in all other subgroups: low FLAIR quality (n=70, AUC=0.61, 95%CI: 0.49-0.74, *P*<0.001), low DWI quality (n=80, AUC=0.62, 95%CI: 0.52-0.72, *P*<0.001), and both DWI/FLAIR low quality (n=180, AUC=0.73, 95%CI: 0.66-0.80, *P*<0.001). All other pairwise comparisons were not statistically significant.

### Supplemental Figure S2: Effect of sequence quality on model performances in the derivation cohort

| **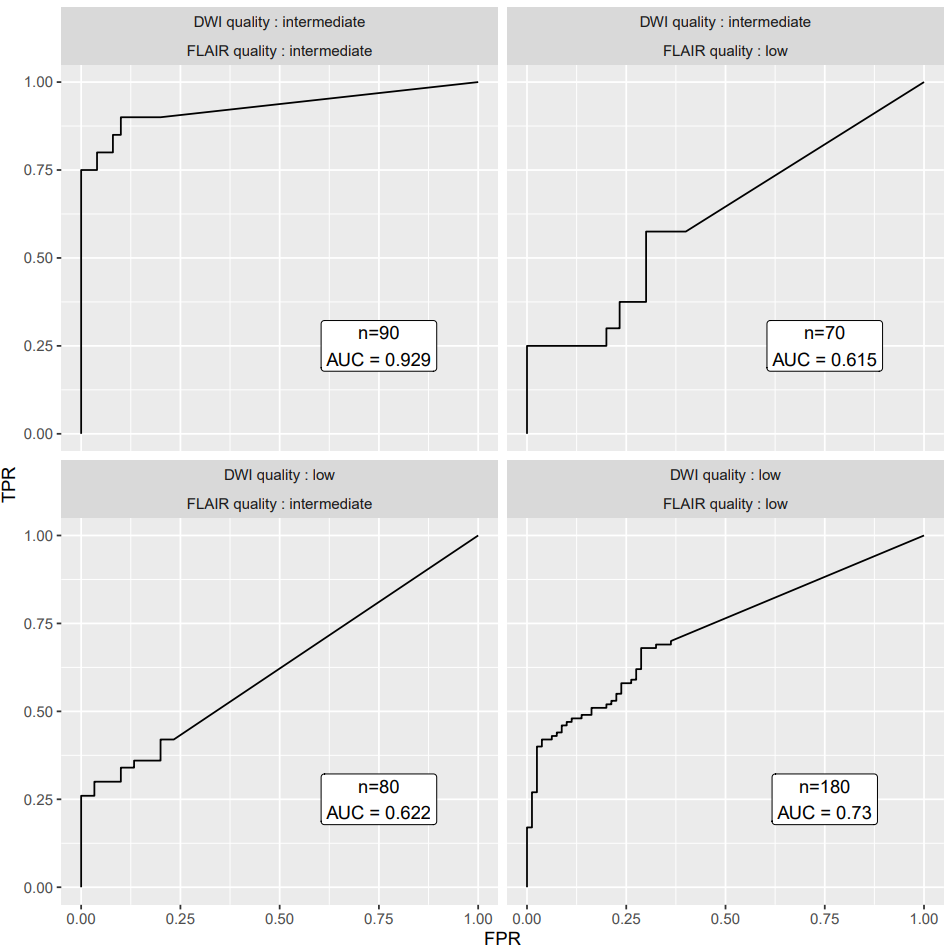**  FPR: False positive Rate. TPR: True Positive Rate. |
| --- |

### Subjects excluded from validation cohort

In the validation cohort, patients excluded for technical issues could not be analyzed. Patients with hemorrhage, no DWI lesion, and extended DWI lesion were studied.

Regarding hemorrhage (n=68), we hypothesized that the model should predict hematoma and hemorrhagic transformations with a high FVA-index, as the training data did contain hemorrhagic transformations on day-1 MRIs. In these patients, median FVA-index was 48 (IQR 26.5-100). After applying the 0.5 threshold, 65/68 (96%) subjects were correctly classified (Supplemental Figure S3, A). On 3 misclassified patients, 2/68 (3%) were subarachnoid hemorrhage, and 1/68 (1%) was a deep brain hematoma on low quality DWI.

Regarding subjects without DWI lesion (n=147), we expected the FVA-index to be near 0 as no stroke should be detected. After applying the 0.5 threshold, 146/147 (99%) subjects were correctly classified. The misclassified patient had a choroid plexus xanthogranuloma which was mistaken for a stroke. Regarding subjects with extended DWI lesion (n=35), in the absence of clear ground truth, we used the initial on-site labeling as ground truth. MRIs had been labeled as DWI-FLAIR mismatch in 6/35 (17%). After applying the 0.5 threshold, 32/35 (91%) subjects were correctly classified. Among the classification error, 2/35 (6%) were False Negatives (DWI-FLAIR mismatch with FVA-index > 0.5) with partial FLAIR visibility on the visualization map, and 1/35 (3%) was a False Positive (no DWI-FLAIR mismatch with FVA ≤ 0.5). (Supplemental Figure S3, B).

### Supplemental Figure S3: Example of model prediction on excluded patients

| 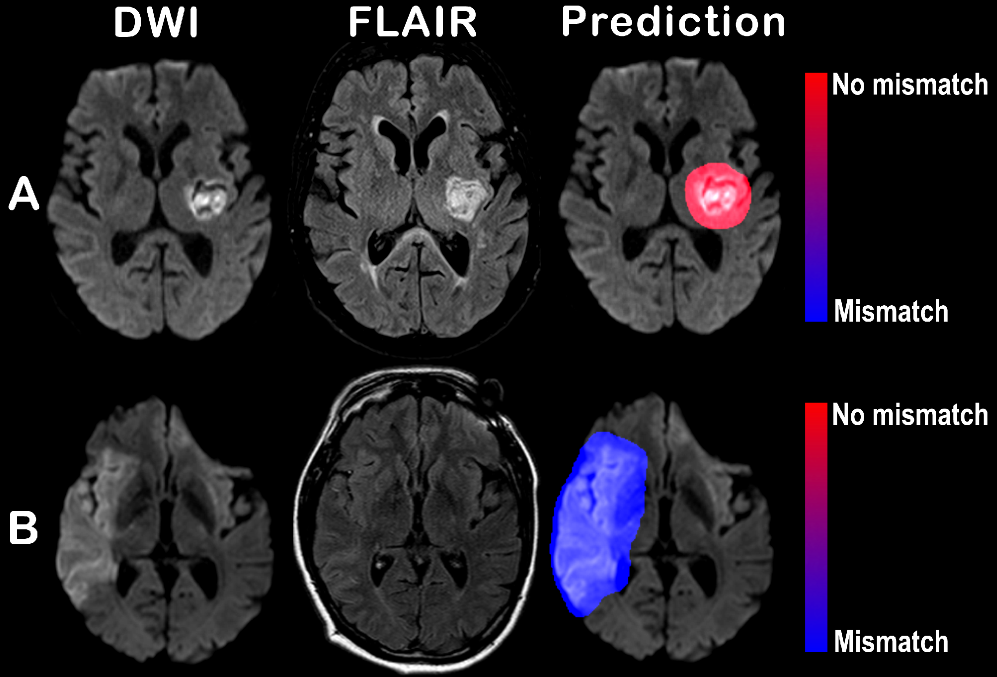  In A, a 56-year old woman presented a left deep brain hematoma on DWI (left column) and FLAIR (middle column). FVA model predicted “No mismatch” (right column) (FVA-index=32).  In B, a 53-year old man presented an extended right middle cerebral artery infarct (>2/3 of vascular territory, left column). Ground-truth prediction was “DWI-FLAIR mismatch” (middle column) and the FVA model accordingly predicted a DWI-FLAIR mismatch (right column) (FVA-index=0). |
| --- |

## Software deployment and performance

The 10 trained models were exported into Open Neural Network eXchange (ONNX) format (https://onnxruntime.ai/). This format allows safe transfer of models and easy deployment on various platforms, as well as GPU or CPU inference.

The inference python script was encapsulated into a Docker container (https://www.docker.com/). The script performed the following tasks: 1/ scanning a directory for b0 and b1000 NIFTI files; 2/ normalization of b0 and b1000 data; 3/ stacking 7-slices for 2.5D inference; 4/ inference on the 10 pretrained models; 5/ rescaling inferred maps into original geometry; 6/ computing RGB heatmap; 7/ computing synthesis PNG file.

Twenty MRIs from the validation cohort were randomly selected to evaluate the inference duration. The performances were evaluated on a consumer laptop (Intel core i7-13700, 2100MHz with 32 GB RAM; GPU acceleration: NVIDIA GeForce RTX 4070 Laptop with 8GB VRAM), and mean time for inference was 55s ± 16s (details in Supplemental Table S4).

The performance was intentionally evaluated using non-professional equipment, in order to show that a baseline < 1min inference was possible on a minimal configuration. Future enhancements could include large-scale deployment on hospital servers, preloading models into VRAM for faster access, and implementing multithreading to optimize the inference process.

|  | Mean time in seconds (±SD) |
| --- | --- |
| 1. Loading script | 2.18 ± 0.92 |
| 1. Scanning directory | 0.95 ± 0.62 |
| 1. Normalization | 4.34 ± 0.88 |
| 1. Stacking slices | 1 ± 3.25 |
| 1. Inference on GPU | 45.14 ± 11.55 |
| 1. Rescaling | 0.51 ± 0.19 |
| 1. RGB heatmap computing | 0.26 ± 0.19 |
| 1. Synthesis PNG computing | 1.15 ± 0.41 |
| Total time | **55.52 ± 16.01** |

**Supplemental Table S4: Detailed duration of each inference step**

# Supplemental references

1. Benzakoun J, Deslys M-A, Legrand L, et al. Synthetic FLAIR as a Substitute for FLAIR Sequence in Acute Ischemic Stroke. Radiology 2022; 303: 153–159.

2. Garyfallidis E. dipy: Diffusion MRI utilities in python, https://dipy.org (accessed 15 July 2021).

3. Pavel I. Segmentation Models Pytorch, https://github.com/qubvel/segmentation_models.pytorch/tree/master?tab=readme-ov-file#citing (2019, accessed 25 September 2024).

4. Chen L-C, Zhu Y, Papandreou G, et al. Encoder-Decoder with Atrous Separable Convolution for Semantic Image Segmentation. Epub ahead of print 22 August 2018. DOI: 10.48550/arXiv.1802.02611.

5. Tan M, Le QV. EfficientNet: Rethinking Model Scaling for Convolutional Neural Networks. Epub ahead of print 11 September 2020. DOI: 10.48550/arXiv.1905.11946.

6. Solovyev R, Kalinin AA, Gabruseva T. 3D Convolutional Neural Networks for Stalled Brain Capillary Detection. Computers in Biology and Medicine 2022; 141: 105089.

7. Hamon G, Legrand L, Hmeydia G, et al. Multicenter validation of synthetic FLAIR as a substitute for FLAIR sequence in acute ischemic stroke. Eur Stroke J 2024; 10: 23969873241263418.

8. López V, Fernández A, Herrera F. On the importance of the validation technique for classification with imbalanced datasets: Addressing covariate shift when data is skewed. Information Sciences 2014; 257: 1–13.

9. Liu C-F, Hsu J, Xu X, et al. Deep learning-based detection and segmentation of diffusion abnormalities in acute ischemic stroke. Commun Med 2021; 1: 1–18.
